# Impact of Chagas Disease in Bolivian Immigrants Living in Europe and the Risk of Stigmatization

**DOI:** 10.1155/2014/514794

**Published:** 2014-02-27

**Authors:** Rafael M. Ortí-Lucas, María C. Parada-Barba, José E. de la Rubia-Ortí, Alejandra Carrillo-Ruiz, María Beso-Delgado, An L. D. Boone

**Affiliations:** ^1^Servicio de Medicina Preventiva, Hospital Clinico Universitario de Valencia, Avenida Blasco Ibáñez 17, 46023 Valencia, Spain; ^2^Universidad Católica de Valencia San Vicente Mártir, Valencia, Spain

## Abstract

*Background.* The prevalence of Chagas disease in endemic countries varies with the kind of vector involved and the socioeconomic conditions of the population of origin. Due to recent immigration it is an emerging public health problem in Europe, especially in those countries which receive immigrant populations with a high prevalence of carriers. The study reviews the impact of the disease on Bolivian immigrants living in Europe, the preventive measures and regulations applied in European countries, and their repercussion on possible stigmatization of certain population groups. *Methods.* The Bolivian immigrant population resident in 2012 was estimated and the affected population in different European countries was calculated with data on carrier prevalence that were recently published. The preventive measures and regulations available in Europe were also reviewed. MEDLINE-PubMed, GoPubMed, and Embase were consulted for the literature review. *Results.* The Bolivian immigrant population has the highest prevalence of Chagas carriers (6.7%–25%) compared to the overall Latin American population (1.3%–2.4%). Only in Spain, France, Belgium, UK, Portugal, Italy, Switzerland, The Netherlands, and Germany, preventive measures are applied to this population. The established regulations are insufficient and completely different criteria are applied in the different countries and this could reflect a certain degree of stigmatization.

## 1. Introduction 

American Trypanosomiasis or Chagas disease is an endemic disease mainly in Central and South America. It is a zoonosis caused by the flagellated protozoan *Trypanosoma cruzi* (*T. cruzi*). In endemic areas, the primary route of transmission is vector borne, usually by triatomine bugs of the Reduviidae family, subfamily Triatominae. It may also be transmitted orally, parenterally, by accidental exposure to contaminated material, by transplantation of organs and/or tissues, via blood transfusion, and vertically from an infected mother to her newborn child. The principal vectors involved in the transmission are grouped into three genera (*Triatoma*, *Rhodnius*, and *Panstrongylus*) highlighting five species with a specific geographical distribution: *Triatoma* infestans is found mainly in southern South America; *Rhodnius prolixus* in the north of South America and Central America; *Triatoma dimidiata* as the previous and in Mexico; *Panstrongylus megistus* in the region from the south-western United States to central Argentina, Brazil, and Paraguay; and *Triatoma brasiliensis* in Brazil and the Amazon basin [1].

The geographical distribution determines in part the clinical symptoms and depends basically on the vector involved. Probably as a consequence of internal migration from rural areas to big cities or migration between different regions within the same country, the two main clinical disorders, cardiac and digestive, are overlapping increasingly. The involvement of different vectors may probably explain the predominance of cardiac symptoms (arrhythmias, focal myocarditis, and different degrees of heart failure) in Bolivian Chagas carriers, especially from the Cochabamba region [2], and predominance of digestive symptoms (anorexia, nausea, vomiting, and diarrhea or chronic constipation and, in more advanced phases, megaintestine such as megaoesophagus and megacolon) in carriers from some regions such as the Argentinian and Bolivian Chaco [3].

The geographical distribution, along with other socioeconomic and cultural factors, could also explain the risk of occurrence of cases in endemic regions and could partly explain the large variability of prevalence in endemic areas, from 1.2% in Ecuador to 50–60% in parts of Bolivia and the Argentinian Chaco [3, 4]. Comparing endemic countries, the prevalence stands out in Bolivia, followed by Argentina, Paraguay, Colombia, Ecuador, and Brazil.

According to the WHO/PAHO, between 15 and 18 million people were parasitized in the 1990s and about 25% of the Latin American population was at risk for Chagas disease, demonstrating it is a serious problem for public health and the socioeconomic development of these countries. The effort made in recent years to control endemic disease has reduced the number of infected individuals to about 6 million, although with a relative increase of vertical transmission and transmission by transfusion. Nevertheless, the infestation is not just limited to this continent and cases were identified in nonendemic areas such as North America, Australia, Japan, and Europe, where it can be considered an emerging disease [5–7]. The prevalence in these countries depends mainly on the proportion of immigrants from endemic areas. Because of human migration, many Latin American immigrants arrived in Europe, especially in Spain, Italy, and Switzerland. As to be expected, the distribution of the prevalence of carriers in Europe is determined by the prevalence in the country of origin. Therefore, the highest prevalence is found in Bolivian immigrants living in Spain [8–10].

In nonendemic countries, public health initially focused on the detection of imported cases that could present complications of the disease. In recent years, however, new native cases have been detected as a result of blood transfusions in the US and Canada [11], Spain [12, 13], and Italy [4]; liver, bone marrow, and cord blood transplants in Spain [14, 15]; and mother to child transmission in Spain [16–18], Romania, Switzerland, Italy [19], and in some regions of the US, where the incidence reached up to 10% [20]. As a consequence, the situation is starting to be considered a serious public health problem, especially in some European countries like Spain.

The growing interest in prevention and treatment of Chagas disease comes across difficulties to diagnose the disease in nonendemic countries. Low epidemiological suspicion, lack of awareness, and nonspecific clinical manifestations in the acute phase (fever, fatigue, swollen glands, and chest pain) make diagnosis by primary care physicians difficult. Moreover, the existence of an intermediate asymptomatic phase can make the disease go undetected for several years or even a lifetime in individuals with positive serology.

To assess the impact of transmission and to improve knowledge of the true burden of disease [21], screening programs are being implemented using different serological tests. The prevalence among immigrants, especially in those of Bolivian origin, and the difficulties in diagnosing and treating the disease may have an effect on the applied preventive measures.

Since Goffman defined stigma in 1963 as “an attribute that extensively discredits an individual, reducing him or her from a whole and usual person to a tainted, discounted one” [22], several sociological studies have related stigma to physical and mental health problems, reduced life expectancy, low socioeconomic status, and poor access to housing, education, employment, and medical care [23, 24].

In this sense, the limitations in access to specific care, considering or not immigrants for donation of blood or organs, in the early detection of disease carriers, or even in the legal regulation of preventive measures, could reflect a discriminative attitude of the sanitary system and public health towards the social group with most carriers.

The aim of the study is to estimate the population of carriers of Chagas disease in Bolivians living in Europe; to get to know the risk of transmission to newborns, receivers of blood transfusion, organ or tissue transplantation, and the accidentally exposed; to review implemented preventive measures and regulations in different European countries (Table 2); and to look into the potential existence of a differential treatment reflecting stigmatization of carriers, understanding the term stigma as a feature that combines the labelling, separation, and social discrediting suffered by the carrier, making him or her hide the problem, behave differently, and suffer discrimination.

## 2. Materials and Methods

To estimate the Bolivian immigrant population carrying *T. cruzi* in Europe, the resident immigrant population with or without official documentation in 2012 was considered together with the prevalence of carriers recently published in different European countries. Data on the population of Bolivian origin was obtained from direct contact with consulates, embassies, and immigrant associations. To obtain data on the prevalence of Chagas disease in European countries, a bibliographic search of scientific literature published during the period 2008–2013 was conducted. Databases MEDLINE-PubMed, GoPubMed, and Embase were consulted. MeSH and DeCS descriptors were used for the literature search and a search strategy was established based on the following descriptors: *Chagas disease, prevalence, emigrants and immigrants, Latin America, Bolivia, Spain, Italy, Switzerland, France, UK, Germany, The Netherlands, Sweden, Austria, Belgium, Croatia, Denmark, Portugal, Romania, Greece, Ireland, Luxembourg, and Norway.* The following inclusion criteria were applied after the literature review: articles published in the period 2008–2013 in any language whose research included human studies of both sexes and immigrants from endemic countries for Chagas disease and that specify the number of Bolivian immigrants that went spontaneously to blood banks, health centres, or maternity centres for pregnancy control. We excluded studies involving only Bolivian immigrants or studies on immigrants seeking medical care for their disease.

The preventive measures and legal framework in terms of control of blood transfusion, organ donation, and screening of pregnant women in all countries studied were also reviewed. For this purpose, a literature search was conducted in MEDLINE-PubMed and Google for all nonendemic countries in the period 2008–2013.

## 3. Results

Establishing the number of Bolivian immigrants affected by Chagas disease is basically only possible in the geographical zone identified as Latin Europe [31] that includes precisely the countries with major presence of immigrants from Bolivia.

As shown in Table 1, the review of the articles published in the last five years with prevalence data from nonendemic European countries for Chagas disease [19] allows one to know the carrier population of the mentioned disease in Spain, Italy, Switzerland, France, Belgium, and The Netherlands. It should be mentioned that the prevalence data of Switzerland and France are estimates based on administrative data; however, they concur with the figures published in the WHO report on prevention and control of Chagas disease in Europe 2009 [19].

Although, in the rest of European countries where no data are available (Table 1), positive cases for *T. cruzi* [32] were detected and the population from endemic countries is fairly identified, the prevalence of Chagas disease in the Bolivian population is not specified. However, and although the data are not specific, it is known that the prevalence of Chagas disease in the Latin American population in these countries is considerably minor (1.3%–2.4%) [32]. This equally coincides with minor presence of Bolivian citizens.

The variability of legislation regulating the preventive measures against Chagas disease in Europe is large. Existing regulations against Chagas disease in different nonendemic European countries (i.e., countries outside Latin America, with or without exceptional vectorial transmission to humans, where there is population exchange with Latin America) are described in the report “Control and prevention of Chagas disease in Europe" published by WHO in 2010 [19]. A review of the legislation to date was also carried out to detect any changes occurred.

Preventive measures regarding the donation of blood for transfusion, donation of organs and tissues for transplantation, and screening in pregnant women to prevent vertical transmission were analysed.

The first official reference on Chagas disease at European level is made in the European Commission Directive 2004/33/EC [30] which defines exclusion criteria for blood donation from donors who were affected with infectious parasitological diseases, including Chagas disease. In 2006, a new directive, 2006/17/EC, [33] was published on the donation and control of human tissues and cells, which referred to Chagas disease. The directive is related to the screening of donors based on their epidemiological history and travels to endemic areas. These directives are met only in some European countries.

In Spain, since the implementation of the Royal Decree 1088/2005 [34–36], a systematic screening is being carried out of donors born in endemic areas, with mothers born in endemic areas and those who received blood transfusions in endemic areas. In addition, travellers are excluded from donating during six months and after this period serology is performed [37]. To prevent vertical transmission, systematic screening of pregnant women at risk is only performed in the Valencian Community and Catalonia. In the Valencian Community this is regulated by the Circular Letter 3/2007 of the General Direction of Public Health [35] and in Catalonia by the protocol governing screening and diagnosis of Chagas disease in Latin American pregnant women and their infants [38].

Measures to prevent transmission through blood transfusion and transplantation of tissues and organs in France resemble the measures implemented in Spain. There is a national regulation [38] to control blood transfusion according to which a systematic screening of donors born in endemic areas is performed; temporary residents are excluded for four months, after which serological tests are performed. Those with mothers born in Latin America undergo systematic serological screening. Regarding the control on organ and tissue transplantation, Directive 2006/17/EC is partly included in the French law, but it is not fully implemented. There is no systematic screening of pregnant women at risk.

In Belgium, a similar screening is performed only in the Flemish Community [39] although it is not legislated. In the rest of the country, donation of Latin Americans is not accepted and travellers are excluded temporarily. There is no systematic screening of pregnant women at risk at national level.

In the UK, by means of a questionnaire previous to donation, donors at risk are defined as those born in endemic areas, with mothers born in endemic areas, who received transfusions in endemic areas, and those who spent more than four weeks in endemic areas [40]. Those considered at risk are excluded from donation for 6 months and then a serological screening test is performed, depending on availability of the test. Where no test is available, donation of people at risk is not accepted. There is no systematic screening of pregnant women at risk at national level.

In some countries like Germany, Italy, The Netherlands, and Switzerland, no systematic serological tests are performed for the detection of Chagas disease in blood banks. Through a questionnaire, the presence or absence of the disease is determined and travels to endemic areas are asked about. People with known Chagas disease are excluded from donation and, depending on the country, travellers on a temporary basis. Systematic screening of pregnant women at risk is only performed in certain cities of some of these countries (Negrar, Bergamo, and Florence in Italy and Geneva in Switzerland).

In Portugal, only a blood bank in Lisbon performs serological screening of people at risk. No systematic monitoring is carried out on pregnant women. In Austria, Luxembourg, and Norway, there are no policies on the control of Chagas disease in blood banks, for transplantation or in pregnant women at risk. No information is available on the policies in Croatia, Denmark, Greece, Ireland, Romania, and Sweden.

There is no specific regulation in any country about accidental exposition to biologic material contaminated with *T. cruzi*.

## 4. Discussion

The global distribution of Chagas disease has recently changed significantly, primarily due to migration [41]; a lot of the people living in endemic areas have been migrating to Europe. The range of countries that are receiving these immigrants is quite large and with substantial socioeconomic diversity [7]. Three European countries (Spain, Italy, and Switzerland) are above average, and of these three, Spain is the one receiving most of them.

Amongst the nationalities of origin, Bolivian immigrants are the most outstanding and this fact has made Bolivian citizens the target population of this study. It has to be mentioned that their home country is making a lot of progress in controlling Chagas disease. The Bolivian government has issued a decree declaring Chagas disease a national priority (Law no. 3374-2006; http://www.leyes.biz./ *Leyes bolivianas*). The National Chagas Program is playing a very important role by diagnosing and treating the population in Bolivia; the disease is being treated without any kind of health care discrimination.

The prevalence of Chagas disease varies greatly even within the same country. For example, as mentioned in two recently published papers [9, 10], there are differences among some Spanish cities, that is, Valencia and Barcelona. Migration patterns of people from different regions in Bolivia might also have an influence, due to the fact that Bolivians who live in a particular area of the country are more inclined to move to a specific community or city in Spain. There is also a difference in prevalence if measured in the entire Bolivian population or only in pregnant Bolivian women.

The great diversity of countries where Bolivian immigrants settle could play an important role at the level of stigmatization and social isolation, due to the fact that health legislation and legal treatment are strikingly different between these countries and even between the communities within the same country. It is also known that the socioeconomic situation of a patient has an effect on morbidity and mortality [8] in certain diseases and it is to be expected that immigrants are particularly vulnerable to this fact. Although some expert opinions give little importance to this [9], recent studies have found a link between mental health and individual behavioural and social adjustment in the around 80,000 Latin American immigrants in Europe affected by Chagas disease. They have highlighted the importance of these factors [10] and advised educational policy interventions in these European countries in a totally different way from that in which they are currently being addressed.

According to the latest survey on immigration by the National Institute of Statistics (INE) in 2007, in the Valencian Community, 10.21% of all immigrants migrated because of lack of employment, 5.93% to get a better job and 4.90% to improve their quality of life, which makes a total of 21.03% that moved for economic reasons [42]. In the case of Bolivian immigrants and those from other Latin American countries, there are a high number of undocumented immigrants. Their socioeconomic situation could be described as more precarious and this is especially reflected in the labour market, where a lot of women end up working in domestic services and many men in construction [43].

Regulations on blood and organ donation and preventive and therapeutic health care vary greatly among the countries where this population has migrated. This could contribute to the feeling of isolation and exclusion of these individuals, something that presumably has a negative effect on the development of the disease, especially in the case of heart disease.

This could also be seen reflected by the existing variability in European countries concerning the handling of blood and organ donors and the differential application of preventive and therapeutic care.

It cannot be determined to what extent potential stigmatization is due to their low socioeconomic status, to the fact of being immigrants, or to the high prevalence of carriers of the disease in Bolivian immigrants, but all these factors could have an influence.

As mentioned before, this fact has not been sufficiently dealt with nor analysed scientifically, and we believe it can be an important issue for future studies. Chagas disease requires a multidimensional approach with strategies and programs that include prevention, medical care, control, and integration. These programs should be designed at European level and should take into account the social and cultural circumstances of this population that have practically no choice but to leave their country and that, in most cases, do not work in high level jobs.

It has to be noticed that the profile of endemic Chagas disease carriers has been changing in recent years. The idea that the disease is exclusively related to poverty should evolve because endemic areas are not just rural and impoverished. At present and due to migration from rural areas to cities, urban population is also affected by the disease and therefore high prevalences are recorded in the big cities of America and in countries where there is no vector. Similarly, the typical precarious housing is beginning to give way to brick houses with tile roofing or buildings, a fact that makes some people do not even know the vector nor the disease.

The social perception of the disease should be changed, to avoid people refusing to get tested. If this does not change, patients may be stigmatized and socially condemned. It should be accepted that there is no real risk to be contaminated from this disease and endemic population should not be isolated as the transmission routes can be easily controlled. We have to take into account that the disease cannot be transmitted by sexual contact or saliva. Concerning the vertical transmission route in Spain, a protocol already exists in the Valencian Community and Catalonia and one is being developed in Madrid and Murcia.

Furthermore, the general population should know that nowadays affected patients are being treated. It is also substantial to provide proper training to patient associations as they play an important role in supporting patients. The expert patient programs promote campaigns to increase illness awareness and provide information on the pathology, a fact that is achieving great results.

## 5. Conclusions

As we have observed in this study, a high percentage of Bolivian immigrants living in Spain and Italy are carriers of Chagas disease, being 18,52% and 17,80%, respectively. It is also striking that the prevalence is particularly high in Switzerland and France where it reaches up to 25%. The prevalence of the disease is higher in Bolivians than in other Latin American immigrants and they are usually more poverty stricken.

Large differences exist between the different countries receiving immigrants in terms of preventive measures on transmission by blood transfusion, organ or tissue donation, and vertical transmission. In countries like Spain or Italy, governments are aware of this illness and more measures are applied, contrasting with other countries such as Austria and Luxembourg which do not apply any preventive measures. It is also noticeable that there are differences between regions within the same country. Some regions, that is, Valencia and Catalonia in Spain and Negrar, Bergamo, and Florence in Italy, do incorporate preventive measures.

The lack of consensus on preventive measures and legislation could reflect and at the same time increase the risk of stigmatization of this population, which could have a negative influence on the development of the disease and its symptoms. It could have its effect on a population that already combines disfavoring characteristics of being immigrants, having a low socioeconomic status, and being carrier of an emerging disease in our environment. We believe that more studies should be carried out to investigate these issues.

To avoid the risk of stigmatization, the existing different policies should be unified and health authorities should provide more information about the disease to immigrants, health workers, and the population in general. In this way, society may be more conscious that the actual risk of transmission in Europe is very low if appropriate precautions are taken.

## Figures and Tables

**Table 1:** Estimation of the Bolivian immigrant population infected with *T. cruzi* residing in European countries.

Country	Immigrant population of Bolivia (*n*)*	Prevalence (%)	Affected population (*n*)
Spain	199.080	18.52^†^	36.867
Italy	40.080	17.80^‡^	7.134
Switzerland	10.200	25.00^□^	2.550
France	6.050	25.00^+^	1.513
Belgium	2.900	14.80**	429
The Netherlands	2.600	6.75^*β*^	176
United Kingdom	4.000	N.A.	N.A.
Germany	3.000	N.A.	N.A.
Sweden	2.000	N.A.	N.A.
Austria	1.900	N.A.	N.A.
Croatia	1.100	N.A.	N.A.
Denmark	1.350	N.A.	N.A.
Portugal	1.800	N.A.	N.A.
Romania	1.200	N.A.	N.A.

*Data transferred by national statistical institutes, embassies, consulates, and Bolivian associations.

^†^Calculated from data of Piron et al. [25] and Navarro et al. [26].

^‡^Angheben et al. [27].

^□^Jackson and Chappui [28].

^+^Lescure et al. [29].

**WHO [19].

^*β*^Bart et al. [30].

N.A.: not available.

**Table 2:** Preventive measures and legislation of Chagas disease in Europe.

Country	Transmission risk	
	Vertical	Blood transfusion/tissue or organ donation
Spain	Systematic screening of pregnant women at risk only in the Valencian Community and Catalonia**	Screening of Latin Americans at risk and travellers (excluded for 6 months)*

France	No systematic screening	Screening of Latin Americans at risk and travellers (excluded for 4 months)*

Belgium	No systematic screening	Screening of Latin Americans at risk and travellers (Flemish Community) Exclusion of Latin Americans and temporarily of travellers (rest of the country)

United Kingdom	No systematic screening	Screening of Latin Americans at risk and travellers only in blood banks that dispose of the serological tests Exclusion in all other blood banks

Portugal	No systematic screening	Screening of donors at risk only in Lisbon

Italy	Systematic screening in Negrar, Bergamo, and Florence	Exclusion of donors with known Chagas disease and temporary exclusion of travellers (3 months)

Switzerland	Systematic screening in Geneva	Exclusion of donors with known Chagas disease

The Netherlands	No systematic screening	Exclusion of donors with known Chagas disease

Germany	No systematic screening	Exclusion of donors with known Chagas disease and temporary exclusion of travellers (6 months)

Austria	No systematic screening	No systematic screening

Luxembourg	No systematic screening	No systematic screening

Norway	No systematic screening	No systematic screening

Croatia	N.A.	N.A.

Denmark	N.A.	N.A.

Greece	N.A.	N.A.

Ireland	N.A.	N.A.

Romania	N.A.	N.A.

Sweden	N.A.	N.A.

*National legislation, **regional legislation, N.A.: no information available.

## References

[1] Galvão C, Carcavallo R, Rocha DD, Jurberg J

[2] (2011). Informe situacional de la epidemiología y el control de la enfermedad de Chagas en Bolivia. *Gaceta Médica Boliviana*.

[3] Biancardi MA, Morena MC, Torres N, Pepe C, Altcheh J, Freilij H (2003). Seroprevalence of Chagas disease in 17 rural communities of “monte impenetrable”, chaco province. *Medicina*.

[4] Torrico F, Alonso-Vega C, Suarez E (2005). Endemic level of congenital Trypanosoma cruzi infection in the areas of maternal residence and the development of congenital Chagas disease in Bolivia. *Revista da Sociedade Brasileira de Medicina Tropical*.

[5] Navin TR, Roberto RR, Juranek DD (1985). Human and sylvatic Trypanosoma cruzi infection in California. *The American Journal of Public Health*.

[6] di Pentima MC, Hwang LY, Skeeter CM, Edwards MS (1999). Prevalence of antibody to Trypanosoma cruzi in pregnant hispanic women in Houston. *Clinical Infectious Diseases*.

[7] Frank M, Hegenscheid B, Janitschke K, Weinke T (1997). Prevalence and epidemiological significance of Trypanosoma cruzi infection among Latin American immigrants in Berlin, Germany. *Infection*.

[8] Lucas RMO, Barba MCP (2009). Prevalence of American tripanosomiasis in pregnant women from a health area of Valencia, Spain. 2005–2007. *Revista Espanola de Salud Publica*.

[9] Muñoz J, Coll O, Juncosa T (2009). Prevalence and vertical transmission of Trypanosoma cruzi infection among pregnant latin american women attending 2 maternity clinics in barcelona, spain. *Clinical Infectious Diseases*.

[10] Barona-Vilar C, Gimenez-Marti MJ, Fraile T (2012). Prevalence of Trypanosoma cruzi infection in pregnant Latin American women and congenital transmission rate in a non-endemic area: the experience of the Valencian Health Programme (Spain). *Epidemiology and Infection*.

[11] Leiby DA (2004). Threats to blood safety posed by emerging protozoan pathogens. *Vox Sanguinis*.

[12] Riera C, Guarro A, El Kassab H (2006). Congenital transmission of Trypanosoma cruzi in Europe (Spain): a case report. *The American Journal of Tropical Medicine and Hygiene*.

[13] de Pedro I P, Martín Rico P, Santamaría S (2008). Caso clínico de Chagas transfusional. *Enfermedades Emergentes*.

[14] Rodriguez A, Perez F, Rodriguez M, Gonzalez M, Flores M (2010). Enfermedad de Chagas en un paciente no procedente de zona endémica tras transplante ortotópico hepático.[Chagas’ disease in a liver patient not born in endemic area]. *Enfermedades Emergentes*.

[15] Forés R, Sanjuán I, Portero F (2007). Chagas disease in a recipient of cord blood transplantation. *Bone Marrow Transplantation*.

[16] Flores-Chavez M, Faez Y, Olalla JM (2008). Fatal congenital Chagas' disease in a non-endemic area: a case report. *Cases Journal*.

[17] Carrilero B, Quesada JJ, Alfayate S, Segovia M (2009). Congenital Chagas disease in a newborn of a Bolivian mother. *Enfermedades Infecciosas y Microbiologia Clinica*.

[18] Solves P, Parada C, Roig R, Hernández MC, Rodríguez R, Prat I (2009). Chagas disease screening in cord blood donors. *Transfusion*.

[19] World Health Organization Control and prevention of Chagas disease in Europe. *Report of a WHO Informal Consultation (jointly organized by WHO headquarters and the WHO Regional Office for Europe)*.

[20] Gilson GJ, Harner KA, Abrams J, Izquierdo LA, Curet LB (1995). Chagas disease in pregnancy. *Obstetrics and Gynecology*.

[21] Basile L, Oliveira I, Ciruela P, Plasencia A (2011). The current screening programme for congenital transmission of chagas disease in Catalonia, Spain. *Eurosurveillance*.

[22] Goffman E (1963). *Stigma: Notes on the Management of Spoiled Identity*.

[23] Clark R, Anderson NB, Clark VR, Williams DR (1999). Racism as a Stressor for African Americans: a Biopsychosocial Model. *The American Psychologist*.

[24] Link BG, Phelan JC (2001). Conceptualizing stigma. *Annual Review of Sociology*.

[25] Piron M, Vergés M, Muñoz J (2008). Seroprevalence of Trypanosoma cruzi infection in at-risk blood donors in Catalonia (Spain). *Transfusion*.

[26] Navarro M, Perez-Ayala A, Guionnet A (2011). Targeted screening and health education for chagas disease tailored to at-risk migrants in Spain, 2007 to 2010. *Eurosurveillance*.

[27] Angheben A, Anselmi M, Gobbi F (2011). Chagas disease in Italy: breaking an epidemiological silence. *Eurosurveillance*.

[28] Jackson Y, Chappuis F (2011). Chagas disease in Switzerland: history and challenges. *Eurosurveillance*.

[29] Lescure F-X, Paris L, Elghouzzi MH (2009). Experience of targeted screening of Chagas disease in Île-de-France. *Bulletin de la Societe de Pathologie Exotique*.

[30] Bart A, Hodiamont CJ, Grobusch MP, van den Brink RBA, Smout AJPM, van Gool T (2011). Chagas disease in the Netherlands: an estimate of the number of patients. *Nederlands Tijdschrift voor Geneeskunde*.

[31] Friedman LM, Perez-Perdomo R (2003). *Legal Culture in the Age of Globalization: Latin America and Latin Europe*.

[32] Basile L, Jansa J, Carlier Y (2011). Chagas disease in European countries: the challenge of a surveillance system.. *Eurosurveill*.

[33] Navarro M, Navaza B, Guionnet A, Lopez-Velez R (2012). Chagas disease in Spain: need for further public health measures. *PLOS Neglected Tropical Diseases*.

[34] Castro E (2006). Transfusión sanguínea y enfermedad de Chagas: iniciativas en Centros de Transfusión de España. *Enfermedades Emergentes*.

[35] Piron M, Maymó R, Hernández J, Vergés M, Portús M, Casamitjana N (2006). Bancos de Sangre y enfermedad de Chagas: estado actual de la legislación española. Resultados preliminares del estudio de la infección por Trypanosoma cruzi en donantes del Banc de Sang i Teixits. *Enfermedades Emergentes*.

[37] Cañavate CC, Izaguirre EC, Brustenga JG (2009). *Enfermedad de Chagas y Donación de Sangre*.

[38] Generalitat de Catalunya (2010). *Protocolo de Cribado y Diagnóstico de la Enfermedad de Chagas en Mujeres Embarazadas Latinoamericanas y Sus Bebés*.

[41] Coura JR, Vĩas PA (2010). Chagas disease: a new worldwide challenge. *Nature*.

[42] Instituto Nacional de Estadística (INE). Encuesta Nacional de Inmigrantes. http://www.ine.es/jaxi/tabla.do?path=/t20/p319/a2007/p02/l0/&file=04014.px&type=pcaxis&L=0.

[43] Montesinos EG (2012). España como destino de la migración boliviana. *Regiones*.

